# QTL mapping of root traits in wheat under different phosphorus levels using hydroponic culture

**DOI:** 10.1186/s12864-021-07425-4

**Published:** 2021-03-11

**Authors:** Mengjiao Yang, Cairong Wang, Muhammad Adeel Hassan, Faji Li, Xianchun Xia, Shubing Shi, Yonggui Xiao, Zhonghu He

**Affiliations:** 1 Institute of Crop Sciences, National Wheat Improvement Centre, Chinese Academy of Agricultural Sciences (CAAS), Beijing, 100081 China; 2 Agricultural Research Institute of Yili, Yili, 835000 Xinjiang China; 3 College of Agriculture, Xinjiang Agricultural University, Urumqi, 830052 Xinjiang China; 4 International Maize and Wheat Improvement Centre (CIMMYT) China Office, c/o CAAS, Beijing, 100081 China

**Keywords:** Genome-wide linkage mapping, Quantitative trait loci, Root traits, SNP array

## Abstract

**Background:**

Phosphorus (P) is an important in ensuring plant morphogenesis and grain quality, therefore an efficient root system is crucial for P-uptake. Identification of useful loci for root morphological and P uptake related traits at seedling stage is important for wheat breeding. The aims of this study were to evaluate phenotypic diversity of Yangmai 16/Zhongmai 895 derived doubled haploid (DH) population for root system architecture (RSA) and biomass related traits (BRT) in different P treatments at seedling stage using hydroponic culture, and to identify QTL using 660 K SNP array based high-density genetic map.

**Results:**

All traits showed significant variations among the DH lines with high heritabilities (0.76 to 0.91) and high correlations (*r* = 0.59 to 0.98) among all traits. Inclusive composite interval mapping (ICIM) identified 34 QTL with 4.64–20.41% of the phenotypic variances individually, and the log of odds (LOD) values ranging from 2.59 to 10.43. Seven QTL clusters (C1 to C7) were mapped on chromosomes 3DL, 4BS, 4DS, 6BL, 7AS, 7AL and 7BL, cluster C5 on chromosome 7AS (*AX-109955164* - *AX-109445593*) with pleiotropic effect played key role in modulating root length (RL), root tips number (RTN) and root surface area (ROSA) under low P condition, with the favorable allele from Zhongmai 895.

**Conclusions:**

This study carried out an imaging pipeline-based rapid phenotyping of RSA and BRT traits in hydroponic culture. It is an efficient approach for screening of large populations under different nutrient conditions. Four QTL on chromosomes 6BL (2) and 7AL (2) identified in low P treatment showed positive additive effects contributed by Zhongmai 895, indicating that Zhongmai 895 could be used as parent for P-deficient breeding. The most stable QTL *QRRS.caas-4DS* for ratio of root to shoot dry weight (RRS) harbored the stable genetic region with high phenotypic effect, and QTL clusters on 7A might be used for speedy selection of genotypes for P-uptake. SNPs closely linked to QTLs and clusters could be used to improve nutrient-use efficiency.

**Supplementary Information:**

The online version contains supplementary material available at 10.1186/s12864-021-07425-4.

## Background

Phosphorus (P) is an important macro-element for ensuring plant development, productivity, and grain quality [1]. P deficiency causes abnormal physiological and biochemical metabolism during critical plant growth stages and resulted yield losses [2]. P as phosphate is immobile in most of the soil types that make its application on the soil surface less beneficial for plants. While efficient uptake of P from deep soil depends on plant’s underground organs [3]. In crops, an efficient root system is crucial for P-uptake. For example, increase in root to shoot ratio in most of the elite cultivars helps to up-take P from deep soil or by growing longer root hairs to exploit the spatial characteristics of soil for maximum nutrient storage in shoots [3, 4]. Therefore, optimization of root and biomass related attributes such as root length, root width, root tips number, root diameter, root biomass and shoot biomass at seedling stage could provide a promising avenue to explore early variations correlated with high P uptake. Genetic diversity for root-related traits under different nutrient conditions has been also considered very important for grain yield enhancement [5, 6]. Therefore, improvement of nutrient up-take through useful variations in seedling root and biomass traits under varied growth conditions could provide a sustainable solution for developing elite cultivars [2, 7, 8].

In wheat, QTLs have been detected for root traits under different P treatments across the 21 chromosomes [9, 10]. But despite the many genetic interactions which have been determined for the seedling biomass and root system architecture traits, still few loci were reported with major effects [11, 12]. Accurate phenotyping of root traits under normal field conditions is difficult, whereas traditional methods such as soil columns and soil cores are time-consuming and laborious for screening of large populations [13, 14]. Artificial systems like sand, germination paper and hydroponic based cultures have been used as proxies for characterization of root traits [4, 15]. Hydroponic culture with digital imaging has given new opportunities to detect number of root traits with different aspects of root development compared with sand culture and germination paper techniques [11]. Moreover, hydroponic technique can be easily applied for rapid and precise screening of large populations to bridge the phenome to genome knowledge gaps. Several common QTL related to root biomass and root system architecture traits have been reported in both hydroponic and field trials conditions [11, 12, 16]. But, there is no report regarding cloning of QTL for P-uptake related root traits or P uptake efficiency yet.

Nowadays, construction of high-density genetic maps has accelerated the accuracy of quantitative genomic analysis. Therefore, it could increase the chance for identification of true loci for complex traits [17]. The 660 K SNP array in wheat has greatly improved the density of genetic maps for QTL analysis compared with the earlier 90 K array [18, 19]. In this study, we have used hydroponic culture-based image pipeline for root phenotyping and 660 K SNPs array for QTL and joint QTL analysis. This will identify useful loci and can help to understand their pleiotropic effect for multiple root morphological and P-uptake related traits during selection. The aims of this study were (1) to evaluate phenotypic diversity of Yangmai 16/Zhongmai 895 derived doubled haploid (DH) population for seedling root and biomass related traits in different P treatments, (2) to identify QTLs for root traits and biomass at seedling stage under low and high P conditions using 660 K SNP array based high-density genetic map and (3).to detect QTL with pleiotropic effect for multiple traits.

## Methods

### Plant materials

The panel of 198 DH lines from the cross of Yangmai 16/Zhongmai 895 were evaluated for root-related traits in hydroponic culture. The female parental Yangmai 16 is a spring wheat cultivar with drought resistance attributes and cover the largest planting area in the middle and lower Yangtze River Region. Zhongmai 895 is a facultative cultivar and widely cultivated in southern parts of the Yellow and Huai Valleys. This has been characterized as high yielding with drought and heat resistance ability, strong roots and early vigor.

### Hydroponic culture and experimental design

Hoagland’s nutrient solution was used [20], and three P levels were kept at zero (control), low and high P contents (KH_2_PO_4_ 0, 0.005, 0.25 mmol/L, respectively). Whereas, levels of KCl in solution were at 0.35, 0.345 and 0.10 mmol/L, respectively in the three treatments to maintain a common nutrient concentration across treatments [21] (Table 1). The experiments were conducted in randomized complete blocks (RCBD), with three replications from March 15 to April 28, 2017, and 30 healthy seeds of each DH line were used for each treatment.

**Table 1:** Nutrient solution ingredients for wheat seedling growth

Ingredient	Concentration (mmol/L)	Ingredient	Concentration (mmol/L)
K_2_SO_4_	0.75	MnSO_4_.H_2_O	0.001
Ca (NO_3_)_2_.4H_2_O	0.25	ZnSO_4_.7H_2_O	0.001
MgSO_4_	0.60	CuSO_4_.5H_2_O	0.0001
FeEDTA	0.04	Na_2_MoO_4_.2H_2_O	0.000005
H_3_BO_3_	0.001	KH_2_PO_4_	0/0.005/0.25
		KCl	0.35/0.345/0.10

The seeds of each DH line were sterilized for 15–20 min in a 10% H_2_O_2_ solution. After rinsing 5–6 times in sterilized water, seeds were placed on moisturized germination paper in glass Petri dishes, crease-side down and left for 36 h in darkness to initiate germination. After the early appearance of germination, seeds were transferred to sand (2 mm diameter) box in a dark environment for seedling growth at 24 °C for 72 h. The sand box was kept under a constant environment room (12 h photo-period: 16 °C day and 13 °C night, light intensity at 400 μmol m^− 2^ s^− 1^ PAR and relative humidity at 70%). Three uniformly sprouted seeds with ~ 5 mm in roots length for each replication were transferred to holes in trays (the seedling was holed with a sponge), and were placed on plastic tanks (660 × 480 × 280 mm) containing 20 L of nutrient solution. The solution was renewed after every 3 days. After 10 days (two-leaf stage) plants were harvested and placed in 30% ethanol prior to imagery for phenotyping.

### Trait measurements

Five root system architecture (RSA) traits, viz. root length (RL), root volume (RV), root diameter (RD), root tip number (RTN), root surface area (ROSA) were captured through images using a scanner (Perfection V700/V750 2.80A; Epson, China). Images were analyzed by using a software RootNav V1.7.5, which was operationally semi-automated [22]. Root biomass-related traits (BRT), including shoot dry weight (SDW) and root dry weight (RDW) were measured after oven-drying for 72 h at 70 °C (mg/plant). Total dry weight (TDW) was estimated as the sum of SDW and RDW, and ratio of root to shoot dry weight (RRS) was measured as ratio between RDW and SDW.

### SNP genotyping and QTL analysis

The DH lines and parent cultivars were genotyped through Wheat 660 K SNP array synthesized by Affymetrix and commercially available from Capital Bio Corporation (Beijing, China; http://www.capitalbio.com). Dataset is available in an online repository named “Dataset Yang et al. at Dryad data bank (please see data sharing link in availability of data and materials section). Genetic map was contacted by Wang et al. [23] from our lab. Briefly, markers with no polymorphisms between parents, severely distorted segregations, and missing rate greater than 20% were removed in the subsequent linkage analysis. Finally, 10,242 markers each representing a bin site were selected to construct the linkage map of Yangmai 16/Zhongmai 895 population., Map was comprised 25 linkage groups, covering all 21 wheat chromosomes. Among them, chromosomes 1B, 2B, 4A and 7A consisted of two linkage groups, and the remaining chromosomes were with only one linkage group. Inclusive composite-interval mapping was used for QTL analysis in IciMappingV4.1 software [24], and averaged data from the three replicates were used for QTL detection. The SNP genotypes of Yangmai 16 were defined as A, and those of Zhongmai 895 as B. Alleles from Yangmai 16 reduced trait values when the additive effects were negative. Kosambi mapping approach was used to convert recombinant frequencies into distance map [25]. Locations of QTLs for the root traits were detected by inclusive composite interval mapping-additive (ICIM-ADD) by using same software as for the QTL analysis. The threshold for significant QTL of each trait was demarcated by 1000-permutations at *P* = 0.05 [26], and minimum LOD score at 2.5 with walking speed at 1.0 cM. Joint QTL analysis for closely linked pleiotropic QTL was performed using the general linear model scripted in lm package of R software. Phenotypic variance explained by each QTL was calculated as demonstrated in Li et al. [27].

### Identification of putative candidate genes

The genes located in the physical intervals of RSA and BRT-associated genomic regions were screened based on the annotations in the wheat reference genome (CS RefSeq v1.0; IWGSC 2018), and those related to growth, development and nutrient mobilization were considered as candidate genes. Gene annotation was retrieved using EnsemblPlant and EMBL-EBI (http://www.ebi.ac.uk/interpro) databases. Gene annotation for putative proteins was performed using BLAST2GO (https://www.blast2go.com/).

### Statistical analysis

Pearson’s correlations analysis among the traits were estimated using averaged data from each replicate. Significance of variances among DH lines, treatments and interactions between genotypes and treatments (G × T) was calculated using following mixed linear model and considered significant at *P* < 0.05.

1 $$ Y= XB+ Z\mu +\mathrm{ge}+\varepsilon $$

where Y is demonstrated as the response from fixed (β) and random (μ) effects with random error (ε), ge is the genotype × environment effect while X and Z illustrate fixed and random effects, respectively. Broad sense heritabilities for all traits were estimated using genotypes as a random effect following [28].

2 $$ {h}^2={\sigma_g}^2/\left({\sigma_g}^2+{\sigma_{gt}}^2/\mathrm{r}+{\sigma_{\varepsilon}}^2/\mathrm{r}\mathrm{t}\right) $$

where *σ*_*g*_^*2*^, *σ*_*gt*_^*2*^ and *σ*_*ε*_^*2*^ represent genotype, genotype (DH line) × P treatment interaction and error variances, respectively, while t is indicated P treatments and r is replicates. The R package was used for all statistical analyses [29].

## Results

### Phenotypic variation and correlations among traits under P treatments

**Fig. 1:**
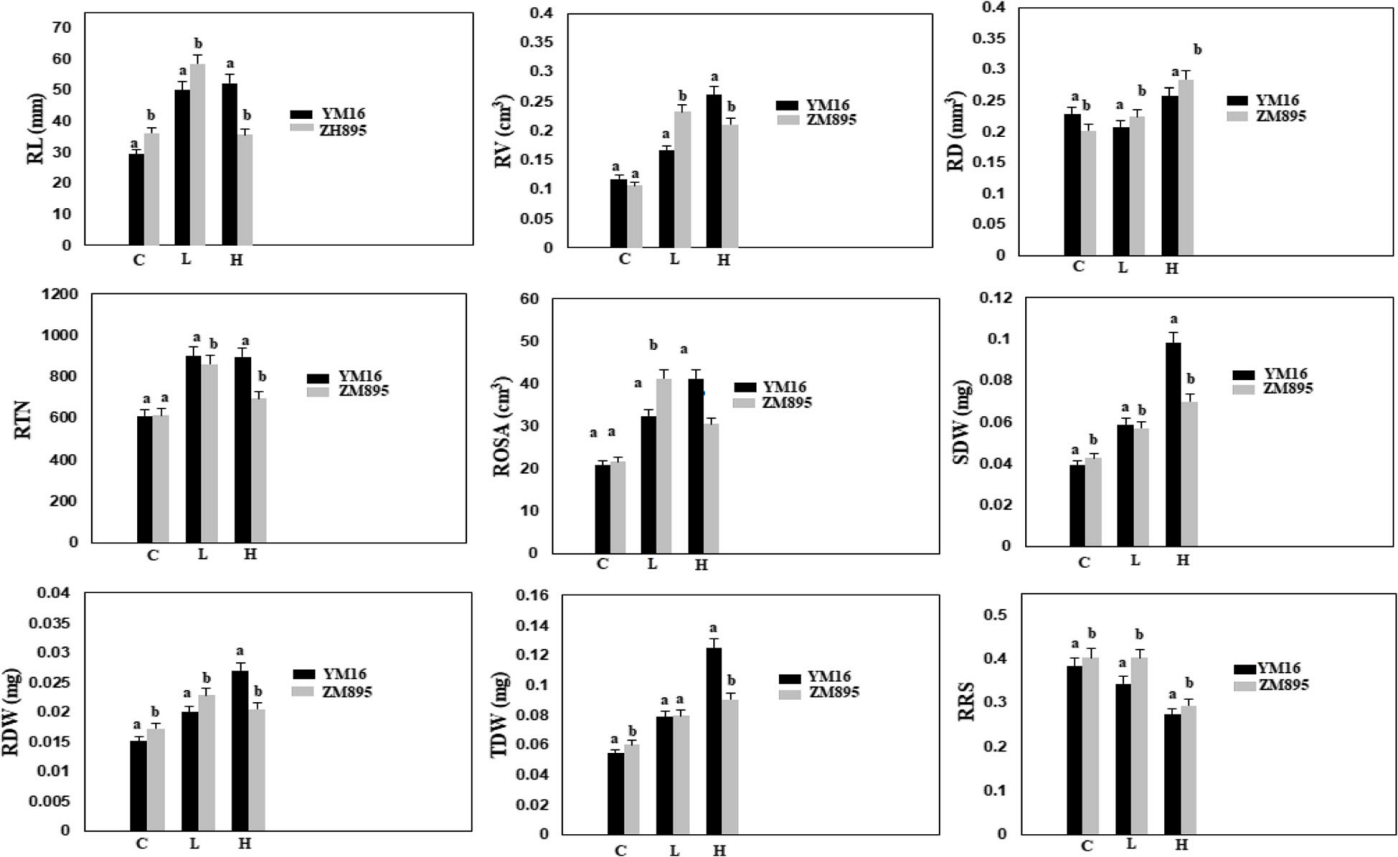
The phenotypic difference between parents Yangmai 16 and Zhongmai 895 under P treatments. Error bars represent standards deviation for each proportion; letters indicate significant differences between the genotypes determined by Duncan tests. Abbreviations: ZM895, Zhongmai 895; YM16, Yangmai 16; C, control; L, low P treatment; H, high P treatment; RL, root length; RV, root volume; RD, root diameter; RTN, root tip number; ROSA, root surface area; SDW, shoot dry weight; RDW, root dry weight; TDW, total dry weight; RRS, ratio of root to shoot dry weight

Data for all nine traits were normally distributed across the three P treatments (Figure S1). Under low P condition, Zhongmai 895 showed higher RL, RV, ROSA, RDW, TDW and RRS, but lower RD, RTN, and SDW than Yangmai 16. Except for RD and RRS in the high P treatment, Yangmai 16 had higher RL, RV, RTN, ROSA, SDW, RDW and TDW than Zhongmai 895 (Fig. 1).

**Fig. 2:**
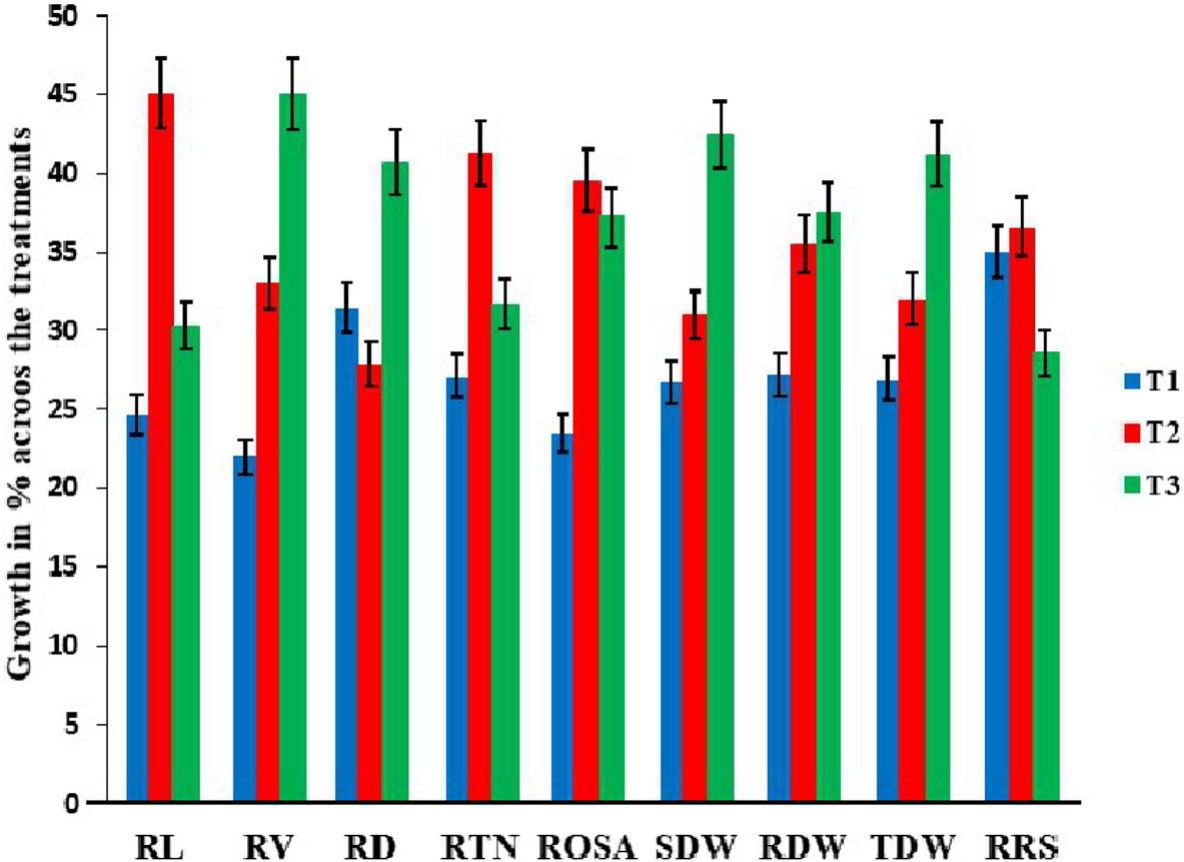
Effects of phosphorus treatment on traits in DH lines. Error bars represent standard deviation for each trait under P treatments. Abbreviations: C, control; L, low P treatment; H, high P treatment; RL, root length; RV, root volume; RD, root diameter; RTN, root tip number; ROSA, root surface area; SDW, shoot dry weight; RDW, root dry weight; TDW, total dry weight; RRS, ratio of root to shoot dry weight

Phenotypic variances among the DH lines were significant (*P* < 0.0001). Transgressive segregations across P treatments were observed for most of traits (Figs. 1, 2; Table 2). The average values of the DH lines for RL, RV, RTN, and RRS were higher than parents in the low P treatment, indicating positive effects for root vigor from both parents (Figs. 1 and 2). Broad sense heritabilities of nine traits were high ranged from 0.76 to 0.91 (Table 2).

**Table 2:** Phenotypic variances for three P treatments in DH lines

Trait	P Level	Min	Max	Mean ± SD	***h***^***2***^	G	T	G × T
						F.value	F.value	F.value
**RL (cm)**	C	18.13	56.86	33.07 ± 10.72	0.90	2.89**	706.75**	1.39**
	L	22.21	98.83	60.51 ± 21.07	0.83	1.53**		
	H	2.01	63.37	40.00 ± 12	0.77	1.09		
**RV (cm**^**3**^**)**	C	0.05	0.23	0.11 ± 0.03	0.86	2.06**	1252.17**	1.46**
	L	0.04	0.34	0.17 ± 0.05	0.81	1.45**		
	H	0.08	0.42	0.22 ± 0.008	0.87	2.28**		
**RD (cm**^**3**^**)**	C	0.16	0.35	0.21 ± 0.03	0.87	2.27**	2274.54**	1.43**
	L	0.14	0.28	0.19 ± 0.02	0.86	2.01**		
	H	0.08	0.42	0.23 ± 0.06	0.88	2.54**		
**RTN**	C	209	1822	716.25 ± 246.7	0.91	3.19**	403.58**	1.34**
	L	183	2477	1090.1 ± 352.41	0.84	1.76**		
	H	184	1593	836.63 ± 341.52	0.82	1.56**		
**ROSA (cm**^**3**^**)**	C	7.25	39.36	21.44 ± 5.24	0.88	2.45**	700.51**	1.36**
	L	7.48	66.38	36.07 ± 10.12	0.82	1.48**		
	H	9.78	66.83	33.99 ± 9.32	0.83	1.68**		
**SDW (mg)**	C	0.02	2.06	0.04 ± 0.009	0.76	1.04	46.72**	1.01
	L	0.02	0.09	0.05 ± 0.01	0.86	2.09**		
	H	0.005	0.12	0.071 ± 0.009	0.88	2.54**		
**RDW (mg)**	C	0.01	0.03	0.02 ± 0.001	0.89	2.69**	451.45**	1.41**
	L	0.01	0.04	0.02 ± 0.001	0.84	1.72**		
	H	0.01	0.12	0.07 ± 0.02	0.89	2.67**		
**TDW (mg)**	C	0.01	2.08	0.06 ± 0.008	0.76	1.05	65.22**	1.03
	L	0.01	0.12	0.07 ± 0.02	0.85	1.88**		
	H	0.01	0.04	0.02 ± 0.001	0.88	2.42**		
**RRS**	C	0.01	0.71	0.38 ± 0.07	0.91	3.34**	180.38**	1.03
	L	0.18	1.04	0.39 ± 0.07	0.83	1.68**		
	H	0.01	0.16	0.09 ± 0.02	0.88	2.50**		

* Significant at *P* < 0.05, ** Significant at *P* < 0.001

Abbreviations: *C* zero control, *L* low P treatment, *H* high P treatment, *RL* root length, *RV* root volume, *RD* root diameter, *RTN* root tip number, *ROSA* root surface area, *SDW* shoot dry weight, *RDW* root dry weight, *TDW* total dry weight, *RRS* ratio of root to shoot dry weight

**Fig. 3:**
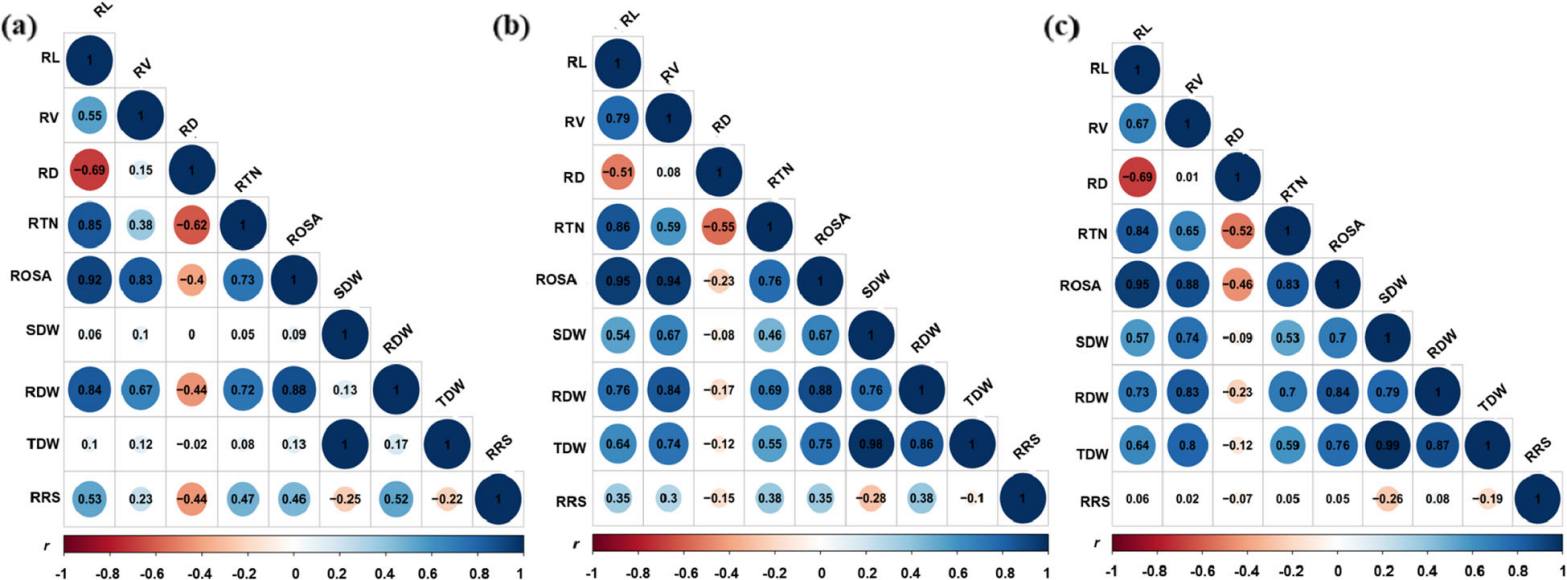
Correlation analysis among the traits under (**a**) control, (**b**) low and (**c**) high P treatment. Intensities of blue colour show degrees of positive and red colour shows degrees of negative correlations; circle sizes indicate low to high significance. Abbreviations: RL, root length; RV, root volume; RD, root diameter; RTN, root tip number; ROSA, root surface area; SDW, shoot dry weight; RDW, root dry weight; TDW, total dry weight; RRS, ratio of root to shoot dry weight

RSA and BRT traits were correlated significantly (*r* = 0.59 to 0.98 at all three P levels) with each other. However, RD was negatively correlated with RL, RTN and ROSA (*r* = − 0.23 to − 0.69) (Fig. 3).

### QTL for root system architecture traits

Nineteen QTL from the three P treatments were identified for RSA traits i.e. RL, RV, RTN and ROSA on chromosomes 1BL, 2BL, 2DL, 3DL, 4BS, 6AL, 6BL (2), 6DS, 7AS (5), 7AL (3) and 7BL (2) (Table 3). Among them 6 QTL were detected for the zero control, and 7 in the low and 6 in the high P treatments with the phenotypic variances explained of 45.1, 48.0, 53.7%, respectively. Seventeen QTL on chromosomes 2BL, 2DL, 3DL (2), 4BS (3), 6AL, 6BL (3), 6DS (2), 7AS (2), 7AL (2) conferred positive additive effects contributed by Zhongmai 895 (Table 3). In the low P treatment, three QTL were identified for each of RL and RTN, explaining 20.7 and 21.7% of phenotypic variances, respectively. RL and RTN were co-located on chromosomes 6BL, 7AL and 7BL. Zhongmai 895 possessed the positive alleles on 6BL and 7AL for increased phenotypic values (Table 3).

**Table 3:** QTLs for root system architecture and root biomass-related traits identified under three P treatments

Trait	Treatment	QTL^**a**^	Marker interval	Physical interval^**b**^ (Mb)	Genetic position^**c**^ (cM)	LOD^**d**^	PVE ^**e**^ (%)	Add ^**f**^
**RL**	**C**	*QRL.caas-1BL*	*AX-10928674* - *AX-94446430*	671.33–688.38	2.5	3.13	5.56	−18.89
		*QRL.caas-7AS.1*	*AX-110432090* - *AX-109345074*	20.95–22.87	0.97	3.51	6.38	19.78
		*QRL.caas-6AL*	*AX-86165298* - *AX-109431293*	612.11–613.49	1.17	4.53	8.20	23.51
		*QRL.caas-7AL*	*AX-109966788* - *AX-94819074*	725.54–734.54	4.84	3.37	6.22	19.54
	**L**	*QRL.caas-6BL*	*AX-109368729* - *AX-95094583*	699.99–711.76	4	4.90	8.61	45.43
		*QRL.caas-7BL*	*AX-95025477* - *AX-109289805*	700.83–705.84	2.35	3.74	6.57	−38.04
		*QRL.caas-7AL*	*AX-109966788* - *AX-94819074*	725.54–734.54	4.84	3.46	5.50	35.19
	**H**	*QRL.caas-7AS.2*	*AX-109955164* - *AX-109445593*	116.62–122.18	1.72	4.72	11.89	30.81
**RV**	**C**	*QRV.caas-6DS*	*AX-10934618* - *AX-94913665*	18.67–25.66	3.45	3.98	8.41	0.01
		*QRV.caas-4BS*	*AX-108815849* - *AX-109491270*	21.48–21.79	2.5	4.90	10.32	0.01
	**H**	*QRV.caas-7AS*	*AX-108763612* - *AX-111263013*	85.44–85.71	1.95	3.49	8.82	0.01
**RTN**	**L**	*QRTN.caas-6BL*	*AX-109368729* - *AX-95094583*	699.99–711.76	4	4.21	7.08	73.56
		*QRTN.caas-2BL*	*AX-86176885* - *AX-94933613*	647.32–647.71	0.35	2.86	4.79	57.39
		*QRTN.caas-7BL*	*AX-95025477* - *AX-109289805*	700.83–705.84	2.35	5.52	9.78	−81.68
		*QRTN.caas-7AL*	*AX-109966788* - *AX-94819074*	725.54–734.54	4.84	2.77	4.64	56.97
	**H**	*QRTN.caas-2DL*	*AX-94959623* - *AX-94821426*	79.54–82.22	1.26	2.74	7.78	45.97
		*QRTN.caas-7AS*	*AX-109955164* - *AX-109445593*	116.62–122.18	1.72	3.84	8.17	48.20
		*QRTN.caas-3DL*	*AX-110036411* - *AX-109917936*	559.77–566.72	2.79	2.99	6.21	42.26
**ROSA**	**H**	*QROSA.caas-7AS*	*AX-109955164* - *AX-109445593*	116.62–122.18	1.72	4.43	10.80	2.24
**SDW**	**H**	*QSDW.caas-6BL*	*AX-109368729* - *AX-95094583*	699.99–711.76	4	3.44	6.80	0.00
		*QSDW.caas-6DS*	*AX-109346183* - *AX-94913665*	18.67–25.66	3.45	2.59	4.95	0.00
		*QSDW.caas-3AS*	*AX-111507145* - *AX-109273188*	15.80–16.63	1.62	3.01	5.99	0.00
		*QSDW.caas-3DL*	*AX-110036411* - *AX-109917936*	559.77–566.72	2.79	4.17	8.21	0.00
**RDW**	**L**	*QRDW.caas-4BS*	*AX-111068079* - *AX-111164540*	32.42–37.86	0.85	7.70	17.70	0.00
		*QRDW.caas-4DS*	*AX-109816583* - *AX-109478820*	16.64–30.66	3.6	3.12	7.14	0.00
	**H**	*QRDW.caas-3AS*	*AX-111507145* - *AX-109273188*	15.80–16.63	1.62	3.08	7.06	0.00
		*QRDW.caas-4BS*	*AX*-*111068079* - *AX-111164540*	32.42–37.86	0.85	3.59	8.12	0.00
**TDW**	**H**	*QTDW.caas-3AS*	*AX*-*111507145* - *AX-109273188*	15.80–16.63	1.62	3.11	7.68	0.00
**RRS**	**C**	*QRRS.caas-4BS.1*	*AX*-*109494015* - *AX-108991675*	97.25–103.06	0.48	6.78	13.59	0.01
		*QRRS.caas-4DS*	*AX*-*109816583* - *AX-109478820*	16.64–30.66	3.6	10.43	20.41	−0.01
	**L**	*QRRS.caas-4BS.1*	*AX*-*109494015* - *AX-108991675*	97.25–103.06	0.48	2.94	6.25	0.01
		*QRRS.caas-4DS*	*AX*-*109816583* - *AX-109478820*	16.64–30.66	3.6	5.55	11.71	−0.01
	**H**	*QRRS.caas-4BS.2*	*AX-108815849* - *AX-109491270*	21.48–21.79	2.5	4.33	9.35	0.01
		*QRRS.caas-4DS*	*AX-109816583* - *AX-109478820*	16.64–30.66	3.6	3.85	8.30	−0.01

^a^ Quantitative trait loci

^b^ Physical positions of SNP markers based on wheat genome sequences from the International Wheat Genome Sequencing Consortium (IWGSC, http://www.wheatgenome.org/)

^c^ Genetic position of closest marker to the identified QTL on linkage map

^d^ LOD value of each QTL

^e^ Phenotypic variance explained by QTL

^f^ A positive sign means the increased effect contributed by Zhongmai 895, whereas the negative effect was contributed by Yangmai 16

Abbreviations: *C* control, *L* low P treatment, *H* high P treatment, *RL* root length, *RV* root volume, *RD* root diameter, *RTN* root tip number, *ROSA* root surface area, *SDW* shoot dry weight, *RDW* root dry weight, *TDW* total dry weight; RRS, ratio of root to shoot dry weight

(Q = QTL) + (caas = Chinese Academy of Agricultural Sciences) + Chromosome = long arm (L) or short arm (S)

### QTL for root biomass-related traits

Fifteen QTL for BRT traits were identified on chromosomes 3AS (3), 3DL, 4BS (5), 4DS (4), 6BL and 6DS (Table 3). Two, 4 and 9 QTL were identified in the zero, low and high P treatments, and explained 34.0, 42.8 and 67.6% of the phenotypic variances, respectively. A stable QTL (*QRDW.caas-4BS*) was identified under both low and high P conditions, explaining 8.1 to 17.7% of the phenotypic variances for RDW. A pleiotropic QTL on chromosome 4DS in interval *AX-109816583 - AX-109478820* (16.64–30.66 Mb) detected in the low P treatment for RDW co-located with 3 QTL in all three P treatments for RRS explained 7.1 to 20.4% of the phenotypic variances (Table 3).

### QTL clusters

**Fig. 4:**
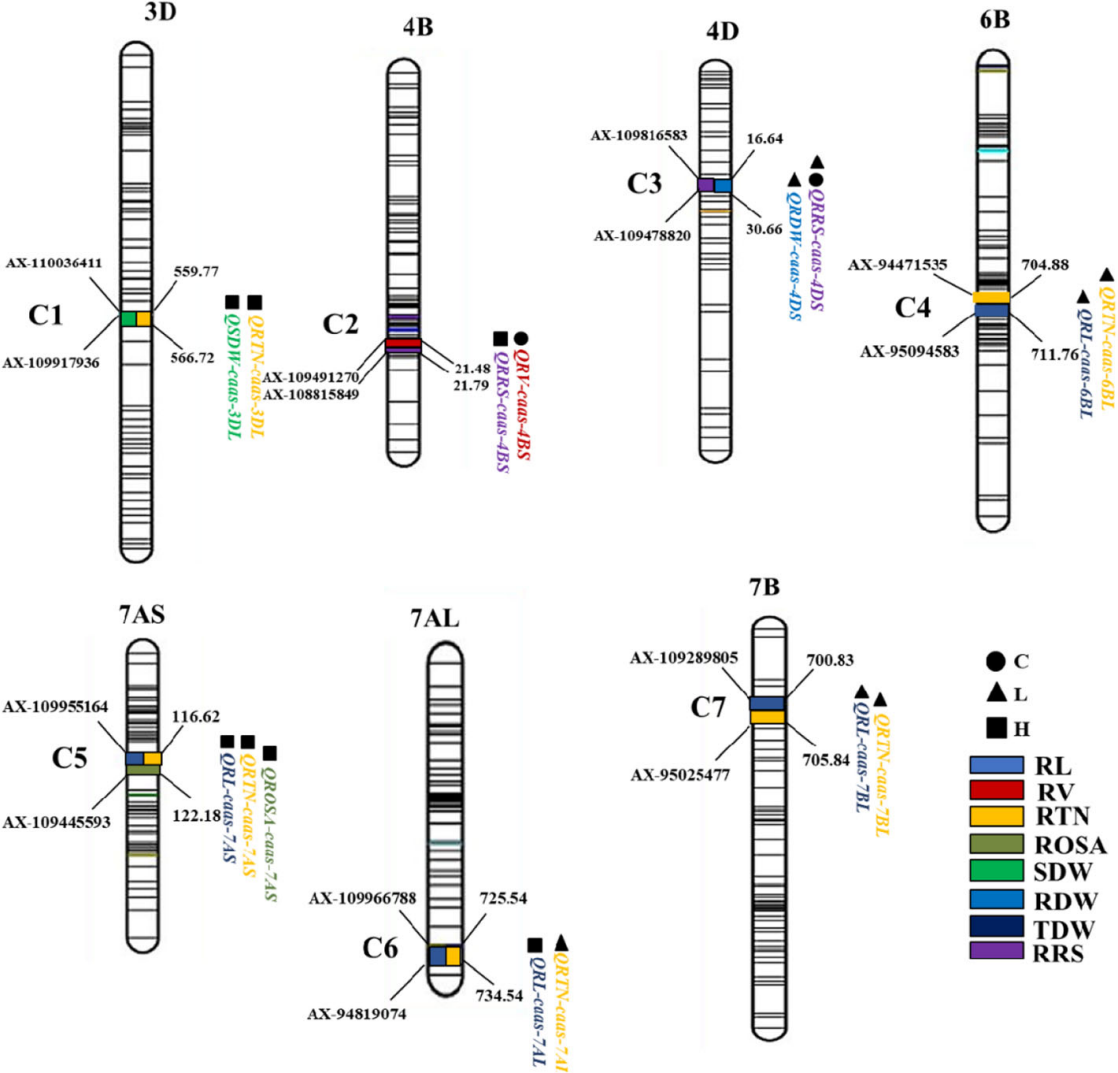
Seven QTL clusters containing pleiotropic loci with closely linked markers and physical positions. Abbreviations: C, control; L, low P treatment; H, high P treatment; RL, root length; RV, root volume; RTN, root tip number; ROSA, root surface area; SDW, shoot dry weight; RDW, root dry weight; TDW, total dry weight; RRS, ratio of root to shoot dry weight

Fifteen QTL were grouped in seven clusters (C1 to C7) for both RSA and BRT traits in all three P treatments. These clusters were identified in the same or close marker intervals (Fig. 4). Genetic regions of these clusters were located on 3DL, 4BS, 4DS, 6BL, 7AS, 7AL and 7BS chromosomes, respectively. Of these, C1 was on 3DL for SDW and RTN under high P treatment. C2 for RV and RRS on 4BS was detected in a 0.31 Mb interval between SNPs *AX-109491270* (21.79 Mb) and *AX-108815849* (21.42 Mb). C3 on 4DS comprised QTL for RDW and RRS. QTL in clusters on 4B (16.64–30.66 Mb) and 4D (32.42–37.86 Mb) were at or very near to reduced plant height gene loci *Rht-B1* (30.8 Mb) and *Rht-D1* (18.9 Mb), respectively. C4, C6 and C7 involved the same traits (RL and RTN) on chromosomes 6BL, 7AL and 7BL. C5 on 7AS (*AX-109955164-AX-109445593*) affected RL, RTN and ROSA, with the favorable allele contributed by Zhongmai 895 (Fig. 4). Joint QTL analysis reveal that, QTLs presented in four clusters. i.e. C3 (RDW and RRS), C4 (RL and RTN), C5 (RL, RTN, ROSA) and C7 (RL and RTN) were identified as pleiotropic QTL under different P levels (Fig. 4 and Table 4).

**Table 4:** Joint QTL analysis results for pleiotropic QTL in clusters

Cluster	Trait	P Level	Chromosome	Marker interval	Physical interval^**a**^ (Mb)	Pr(>|t|)^**b**^	Adjusted R^**2**^
C1	SDW	H	3D	*AX-110036411-AX-109917936*	559.77–566.72	0.02*	0.38
	RTN	H					
C2	RV	C	4B	*AX-109491270-AX-108815849*	21.48–21.79	0.05*	0.12
	RRS	H					
C3	RDW	L	4D	*AX-109816583-AX-109478820*	16.64–30.66	0.15, 0.18	0.18, 0.12
	RRS	C, L					
C4	RL	L	6B	*AX-94471535-AX-95094583*	704.88–711.76	0.7	0.75
	RTN	L					
C5	RL	H	7A	*AX-109955164-AX-109445593*	116.62–122.18	0.68, 0.55, 0.71	0.76, 0.90, 0.74
	RTN	H					
	ROSA	H					
C6	RL	H	7A	*AX-109966788-AX-94819074*	725.54–134.54	0.037*	0.12
	RTN	L					
C7	RL	L	7B	*AX-109289805-AX-95025477*	700.83–705.84	0.8	0.75
	RTN	L					

* Indicates significance at *P* < 0.05

^a^ Physical positions of SNP markers based on wheat genome sequences from the International Wheat Genome Sequencing Consortium (IWGSC, http://www.wheatgenome.org/)

^b^ Significance of t-values at *P* < 0.05

Abbreviations: *C* control, *L* low P treatment, *H* high P treatment, *RL* root length, *RV* root volume, *RD* root diameter, *RTN* root tip number, *ROSA* root surface area, *SDW* shoot dry weight, *RDW* root dry weight, *TDW* total dry weight, *RRS* ratio of root to shoot dry weight

## Discussion

### Significant variations and correlations observed for RSA and BRT

Hydroponic-based rapid phenotyping approach was used to measure for BRT and RSA related traits in DH population. Previous studies identified strong correlations between such laboratory-based experiments and field data [30, 31]. Therefore, use of digital dataset on BRT and RSA can be used to explore phenotypic variations among the DH population under varied nutrient levels. Most of the phenotypes were higher under the high P treatment compared with zero and low P treatments (Fig. 2). High heritabilities and significant genetic variances for root system architecture and biomass traits indicated that these traits could be used as primary selection criteria for enhancement of P uptake and to identify underlying genetics [12, 17] (Table 2). The present results corroborated earlier findings for genetic variances of root-related traits under different nutrient treatments [6, 32, 33].

P deprivation restricts the growth of main roots, while increases the lateral roots elongation with high numbers of root hairs [34]. This phenomenon leads higher ratio of root to shoot that significantly changes the root architecture for greater nutrient up-take [10]. In this study, RSA traits i.e. RL, RV, RTN and ROSA, and BRT traits i.e. RDW and RRS showed higher growth and highly positive correlation between each other at the low P level (Figs. 2 and 3). This trend was contributed by Zhongmai 895, which also had longer roots, high root tip number and high RRS in the low P treatment. High accumulation of SDW under high P conditions and greater RRS under low P were observed in Yangmai 16 (Fig. 1). Zhongmai 895 performed higher as compared to Yangmai 16 across the P treatments, as previously reported elite for high root growth at seedling stage and field-based nitrogen use efficiency [35, 36]. SDW and RRS were negatively correlated across treatments (Fig. 3), although previous work found that low P led to reduced root biomass and had a significant impact on root system architecture traits [4, 17, 37]. A significantly negative correlations ranging from − 0.23 to − 0.69 of RD with RL, RTN and ROSA in all three P treatments indicated a negative association of RD with high P uptake among DH lines (Fig. 3). A similar trend also observed for Zhongmai 895 under high P treatment (Fig. 1). These kind of variations among the population for complex root behavior could be important for genetic dissection of useful loci.

### QTL identified under phosphorus treatments

Significant variances among genotypes allowed to explore genomic regions associated with the observed traits [4]. Several QTL were identified previously using hydroponic culture, which showed a vital role in nutrient uptake and a positive correlation with yield-related morphological traits. Here we identified 34 QTL on 1B, 2B, 2D, 3A, 3D, 4B, 4D, 6A, 6B, 6D, 7A and 7B chromosomes across the P treatments (Table 3). Some QTL were already reported for root system architecture and root biomass-related traits [12, 38]. New loci closely linked with *AX-109273188* (16.63 Mb) on 3AS, *AX-10934618* (18.67 Mb) on 6DS, *AX-109955164* (116.62 Mb) on 7AS, *AX-109109966788* (725.54 Mb) on 7AL and *AX-109289805* (705.84 Mb) on 7BL showed significant influence for root growth under low P-condition.

The genetic diversity for root vigor under low P conditions could potentially improve P acquisition efficiency and described some QTL for BRT under low P conditions [11]. We also detected QTL on 2BL, 4BS, 4DS, 6BL, 7AL and 7BL showing high phenotypic variances for RSA traits i.e. RTN, RRS and RL under low P. (Table 2). Previously, Su et al. [9, 37] evaluated two DH populations in pot and field experiments and reported a common QTL on chromosome 4B associated with shoot biomass and tiller number, was potentially important for P efficiency. Here, QTL in same genomic region of the 4B chromosome were detected for RDW and RRS (Table 3). This QTL was nearly co-located with *Rht* genes for plant height. The *Rht-1* locus on 4B, which was a major factor against lodging as part of the green revolution. Interactions between root-related growth traits and *Rht* genes were reported as important in early vigor and nutrient uptake [11, 39–41]. These reports had demonstrated contrasting impact of *Rht* genes on seedling traits under different nutrient conditions as compare to our findings. In our results, both QTL mapped on 4B (16.64–30.66 Mb) and 4D (32.42–37.86 Mb) in low P corresponding to *Rht-B1b* (30.9 Mb) and *Rht-D1b* (18.9 Mb), respectively, had negative impact on RRS among DH lines in contrast with a previous report [40].

QTL on 2DL and 6BL for RTN, RL and SDW under low P condition were likely to be those which were demonstrated for root related traits under P sufficiency in pot trials [37] and hydroponic culture [4]. Therefore, identification of those QTL influencing root traits for high nutrient uptake under varied conditions could be important for genotypic selection. RL, RTN and ROSA are important traits for nutrient uptake and early seedling vigor. Five QTL on chromosome 7A explained higher phenotypic variances of 4.6 to 11.9% for RL, RTN and ROSA. Four of these QTL detected under high P conditions and negatively influenced the root elongation, RTN and ROSA. Whereas, the QTL on 7AL controlling RTN had a positive role by increasing RTN in low P condition (Table 3). This QTL could play a vital for future crop breeding to improve the P-uptake. Closely linked QTL *QRL.caas-7BL* and *QRTN.caas-7BL* near *AX-109289805* (705.84 Mb) could also be important for early vigor under P deficient conditions, whereas their strong relationship with kernel number per spike was earlier demonstrated by Zhang et al. [42]. SNPs linked with QTL for these traits on 7A and 7B stably detected in different nutrient conditions might be of great value for wheat breeding.

### Putative candidate genes

Putative candidate genes for new QTL identified on 3AS for SDW, RDW and TDW was linked with E3-Ubiquitin ligase gene (TraesCS3A01G030100 14Kb) (Table 5). It had been reported that E3 ubiquitin ligases are involved in lateral root development in monocots and dicots through regulating plant phytohormone biosynthesis, transport and signaling pathways or cell cycle progression. Whereas, Ubiquitin-mediated proteolysis also has a pivotal role in root development, flowering time control and hypocotyl elongation [43]. Based on putative analysis, flanking sequence of SNP linked with QTL on 7AS identified for RL, RTN and ROSA was associated with gene for Gb protein. Previous reports had shown significant role of Gb protein in stress tolerance and plant growth [44]. Furthermore, a gene for transmembrane domain containing protein family was associated with QTL on 7BL for RL and RTN. These genes have been reported for nutrient import through cell membrane, stress tolerance and vegetative growth in *Arabidopsis* [45]. These QTL could play an important role for high uptake of P through alteration in root traits under varied P conditions.

**Table 5:**
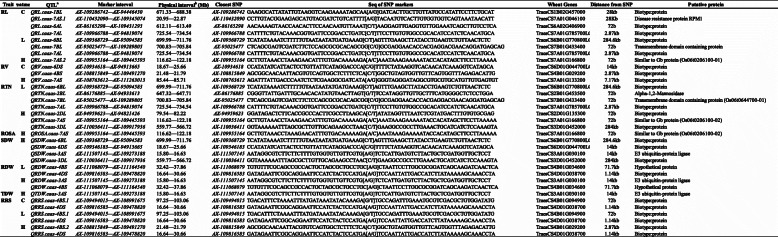
QTL with corresponded candidate genes and putative proteins

^a^ Quantitative trait loci

^b^Physical positions of SNP markers based on wheat genome sequences from the International Wheat Genome Sequencing Consortium (IWGSC, http://www.wheatgenome.org/)

Abbreviations: *C* control, *L* low P treatment, *H* high P treatment, *RL* Root length, *RV* Root volume, *RD* Root diameter, *RTN* root tip number, *ROSA* root surface area, *SDW* shoot dry weight, *RDW* root dry weight, *TDW* total dry weight, *RRS* ratio of root to shoot dry weight

### QTL clusters

Previously high-density physical mapping results demonstrated about 85% of gene expression and also has many cluster distributions in wheat genome that covered 5 to 10% of chromosome regions [46]. In wheat, many QTL clusters were reported in several studies [47–51]. In the present study, seven clusters were identified in three different P treatments (Fig. 4). Clusters C3, C4, C5 and C7 had pleiotropic QTLs for root system architecture and biomass-related traits under different P treatments, whereas QTLs in other clusters contained closely linked multiple genes without pleiotropic effects. Clusters C2 and C3 on chromosomes 4BS and 4DS were linked with reduced plant height genes. Cluster C4 on 6BL had QTLs controlling RL and RTN and previously were reported for thousand grain weight [17]. Cluster C5 on chromosome 7AS (*AX-109955164* - *AX-109445593*) affecting RL, RTN and ROSA was identified for the first time in the present study and SNP linked with this cluster can be used for future wheat improvement. Whereas cluster C7 on chromosome 7BL containing QTLs for RL and RTN was reported for RL and SDW under P-deficient condition. Similar QTL were also identified on 7B chromosome in previous studies [4, 17], but it is not confirmed that QTL in our results are new or co-localization with previously identified loci. (Fig. 4). These results also indicated that QTL identified at seedling stage could be reliable for the selection of yield-related traits measured at maturity [12]. The SNPs tightly linked to QTLs or QTL clusters identified in the present study can be converted to KASP assays and effectively used for MAS to improve nutrient-use efficiency in wheat breeding.

## Conclusions

Thirty-four QTL with significant phenotypic variations for root system architecture and biomass-related traits were identified using high-density genetic map constructed from 660 k SNP array and cost-effective hydroponic-based phenotyping pipeline. Four QTLs on chromosomes 6BL (2) and 7AL (2) identified in low P treatment with positive additive effects from Zhongmai 895, indicating that it could be used as parent for P-deficient breeding. A stable QTL *QRRS.caas-4DS* (16.64—30.66 Mb) was also detected across the three P levels, accounted for 8.4 to 20.4% of the phenotypic variances, which could be used for speedy selection of genotypes for P-uptake. Among the seven QTLs clusters, C5 identified on chromosome 7AS (*AX-109955164*-*AX-109445593*) affecting RL, RTN and ROSA could be vital for improving the nutrient use efficiency of roots under high P condition, whereas, C6 on 7AL under low P condition. Identified chromosome regions, particularly the QTL clusters could be used for molecular marker-assisted selection in future breeding.

## Supplementary Information

**Additional file 1: Figure S1.** Distribution of line and parent means for each trait in three levels of P treatments.

## Data Availability

The datasets are available in the “Dataset Yang et al.” repository at Dryad data bank. Data can be accessed using following link; https://datadryad.org/stash/share/BTR6YCbZX1mr-vH5QojHRYlPHe4uZ5vWSsGmVE2jbPk Citation: Xiao, Yonggui (2021), Dataset Yang et al., Dryad, Dataset, 10.5061/dryad.5hqbzkh54.

## Abbreviations

RSA: Root system architecture
BRT: Biomass-related traits
RL: Root length
RV: Root volume
RD: Root diameter
RTN: Root tip number
ROSA: Root surface area
SDW: Shoot dry weight
RDW: Root dry weight
TDW: Total dry weight
RRS: Ratio of root to shoot dry weight
PUE: Phosphorus use efficiency
DH: Double haploid
QTLs: Quantitative trait loci
LOD: Log-of-odds
