# Supplementary material for: QTL mapping of root traits in wheat under different phosphorus levels using hydroponic culture
Source: BMC Genomics. 2021 Mar 11;22:174. doi: 10.1186/s12864-021-07425-4 (PMC7953759; doi:10.1186/s12864-021-07425-4)
Supplement: Supplementary file 1 — Additional file 1: Figure S1. Distribution of line and parent means for each trait in three levels of P treatments. [file 12864_2021_7425_MOESM1_ESM.docx]

**Supplementary File**

**Fig. S1.** Distribution of DH line and parent means for each trait in three levels of P treatments.

Abbreviations: ZM895, Zhongmai 895; YM16, Yangmai 16; RL, root length; RV, root volume; RD, root diameter; RTN, root tip number; ROSA, root surface area; SDW, shoot dry weight; RDW, root dry weight; TDW, total dry weight; RRS, ratio of root to shoot dry weight.
